# Ocelot Population Status in Protected Brazilian Atlantic Forest

**DOI:** 10.1371/journal.pone.0141333

**Published:** 2015-11-11

**Authors:** Rodrigo Lima Massara, Ana Maria de Oliveira Paschoal, Paul Francis Doherty, André Hirsch, Adriano Garcia Chiarello

**Affiliations:** 1 Departamento de Biologia Geral, Instituto de Ciências Biológicas, Universidade Federal de Minas Gerais, Belo Horizonte, Minas Gerais, Brazil; 2 Instituto SerraDiCal de Pesquisa e Conservação, Belo Horizonte, Minas Gerais, Brazil; 3 Department of Fish, Wildlife, and Conservation Biology, Colorado State University, Fort Collins, Colorado, United States of America; 4 Programa Institucional de Bioengenharia, Universidade Federal de São João Del Rei, Sete Lagoas, Minas Gerais, Brazil; 5 Departamento de Biologia, Faculdade de Filosofia, Ciências e Letras de Ribeirão Preto, Universidade de São Paulo, Ribeirão Preto, São Paulo, Brazil; Clemson University, UNITED STATES

## Abstract

Forest fragmentation and habitat loss are detrimental to top carnivores, such as jaguars (*Panthera onca*) and pumas (*Puma concolor*), but effects on mesocarnivores, such as ocelots (*Leopardus pardalis*), are less clear. Ocelots need native forests, but also might benefit from the local extirpation of larger cats such as pumas and jaguars through mesopredator release. We used a standardized camera trap protocol to assess ocelot populations in six protected areas of the Atlantic forest in southeastern Brazil where over 80% of forest remnants are < 50 ha. We tested whether variation in ocelot abundance could be explained by reserve size, forest cover, number of free-ranging domestic dogs and presence of top predators. Ocelot abundance was positively correlated with reserve size and the presence of top predators (jaguar and pumas) and negatively correlated with the number of dogs. We also found higher detection probabilities in less forested areas as compared to larger, intact forests. We suspect that smaller home ranges and higher movement rates in smaller, more degraded areas increased detection. Our data do not support the hypothesis of mesopredator release. Rather, our findings indicate that ocelots respond negatively to habitat loss, and thrive in large protected areas inhabited by top predators.

## Introduction

Fragmentation and habitat loss are serious threats to tropical forest biodiversity [1, 2] and the Atlantic Forest is no exception [3–5]. The vast majority of remnants (> 80%) in this biome are smaller than 50 ha and 61% of these are more than 25 km from protected areas (PAs), which protect only 9% of the remaining forest and 1% of the biomes’ original area [4]. This biome scenario is inadequate for the long-term conservation of top predators such as jaguars (*Panthera onca*) and mountain lions (*Puma concolor*) [6, 7].

While impacts of forest loss and fragmentation are well documented for large predators [8, 9], the effects on mesocarnivores are less clear. Mesocarnivores are species belonging to the order Carnivora that are neither large nor top predators [10]. They are small or medium-sized species (less than 15 kg); may be solitary to highly social, frugivorous to strictly carnivorous, and have high phenotypic plasticity [10]. These life-history characteristics might allow some species of mesocarnivores to “replace” top predators when such species are absent or declining, altering the food chain (mesopredator release theory; [11]).

The ocelot (*Leopardus pardalis*) is a mesocarnivore in neotropical forests that may thrive in forest patches where top predators are absent or rare [12]. In these circumstances, ocelot might expand its trophic niche in response to a competitive release [12]. Normally, ocelot diets are composed of small mammals (<2.0 kg; [13]), but recent studies suggest that in the absence of top predators, especially jaguars, ocelots take larger prey [14–16]. Ocelots can also prey on other mesocarnivores [17–19] and hunt or harass smaller felines, such as jaguarondi (*Puma yagouaroundi*), margay (*Leopardus wiedii*) and oncilla (*Leopardus tigrinus*) [20, 21]. Together, these findings suggest that ocelots are opportunistic, ecologically plastic and may thrive in fragmented landscapes [22, 23].

However, ocelots may be more sensitive to fragmentation than other mesocarnivores because the species may have high affinity for closed canopy forests [24, 25]. The species is considered vulnerable in fragmented areas outside the Brazilian Amazon, such as the Atlantic Forest [26]. Thus, two opposing forces may be affecting ocelot populations in fragmented landscapes. The abundance of ocelots may be increasing due to mesopredator release or, abundance may be decreasing due to fragmentation and habitat loss. To test these two main hypotheses, and to understand the ecological process driving ocelot population dynamics and conservation status, we estimated ocelot abundance in a range of Atlantic Forest PAs. Specifically we assessed the effects of the amount of habitat (percent of forest cover and reserve size), impact of an invasive domestic species (relative abundance of free-ranging domestic dogs) and presence of top predators (mountain lions and jaguars) on ocelot abundance. We hypothesize a positive relationship between ocelot abundance and reserve size because larger forested areas could support more ocelots [6, 7, 27]. We expect a negative relationship between ocelot abundance and domestic dogs and top predators, because these species are considered potential competitors to ocelots [28, 29].

Camera traps are a common tool used to assess ocelot density [29–33], but few studies have accounted for potential variation in detection probability (*p*). To prevent potential biases caused by such variation, we tested several hypotheses involving factors that may influence detection. We expected that detection probability may vary among the sexes: females may have a higher detection probability than males because they have smaller home ranges that they use more intensively [13]. Alternatively, males travel larger distances [34], and they may be exposed to more cameras than females and thus have a higher detection probability. We expected a trap shy behavioral response in which recapture probability (*c*) of ocelots would be lower than the initial detection probability (*p*) because of the camera flash [35, 36]. We also expected ocelots to be more elusive and restrict their movements in areas with a higher abundance of top-predators or dogs [28, 29]. The number of unpaved roads within a reserve could also influence detection because ocelots often use trails or unpaved roads to move around the landscape [37–39]. We hypothesized that detection probability would be negatively correlated with density of travel routes because we could not survey many routes with our few cameras. Further, detection may be influenced by the location of cameras. Given the known affinity of ocelots for unpaved roads, we expected a positive relationship between detection and proportion of cameras installed on unpaved roads. We also expected a low detection probability in large densely, forested areas (the preferential habitat of the species; [24, 40]), because individuals have more area to explore and may have larger home ranges. Finally, we expected a higher detection probability in dry seasons because ocelots may be more active in the dry season due to resource scarcity [41].

In summary, our main objective is to estimate ocelot abundance and density in six Atlantic Forest reserves in southeastern Brazil, while correcting for factors that may influence detection. We also assess the ability of reserve and individual ocelot variables to explain variation in ocelot abundance and detection. Finally, we compare our estimates with other estimates to assess the current ocelot population status in Atlantic Forest remnants.

## Materials and Methods

### Ethics statement

Sampling was performed under licenses obtained from the State Forest Institute (Instituto Estadual de Florestas—IEF) of the State Parks (UC: 080/10, 081/ 10 and 082/10) and under permission from the responsible (the owner of the land) of the private reserves. Data collection used non-invasive, remotely activated camera traps and did not involve direct contact or interaction with animals.

### Study areas

**Fig 1:**
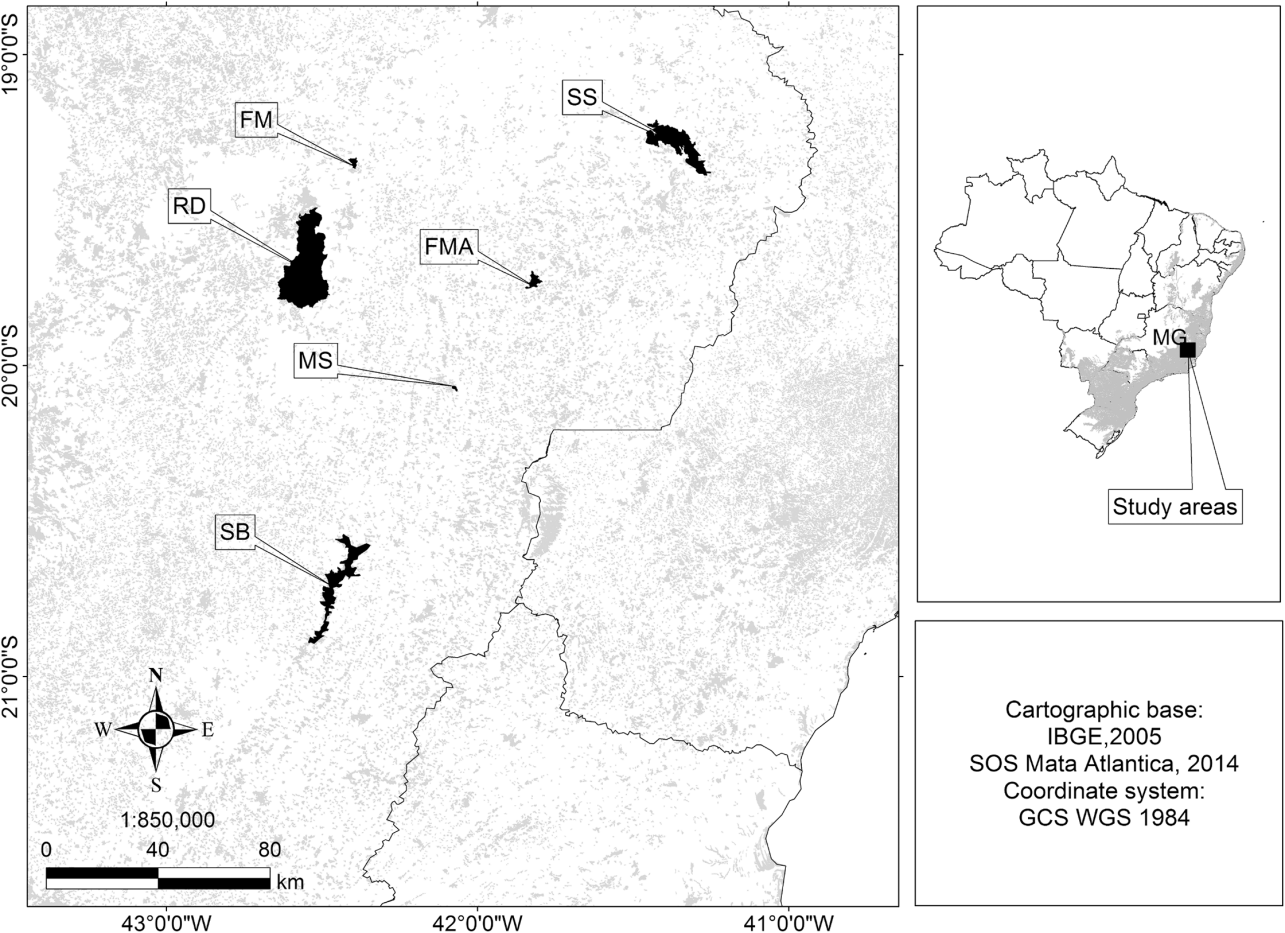
Atlantic Forest reserves sampled for ocelot populations in State of Minas Gerais (MG), southeastern Brazil. FM = Fazenda Macedônia Reserve; FMA = Feliciano Miguel Abdala Reserve; MS = Mata do Sossego Reserve; SB = Serra do Brigadeiro State Park; SS = Sete Salões State Park; RD = Rio Doce State Park. The current distribution of Atlantic Forest remnants are shown in the insert (grey area) as defined by the SOS Mata Atlântica Foundation [47]. The state divisions are from the Brazilian Institute of Geography and Statistics [48].

We sampled six protected areas in the Atlantic Forest located in the State of Minas Gerais, southeastern Brazil (Fig 1). These include one large (> 20,000 ha) and two medium-sized (10,000–20,000 ha) state parks, respectively: Rio Doce (RD), Serra do Brigadeiro (SB) and Sete Salões (SS), and three small (< 10,000 ha) private reserves: Feliciano Miguel Abdala (FMA), Mata do Sossego (MS), and Fazenda Macedônia (FM). Vegetation in all areas is classified as semi-deciduous seasonal forest [42]. Elevation in these areas ranges from 150 m (RD) to 2,075 m (SB) [43] and the climate is classified as humid tropical in SB and semi-humid in the other PAs [44]. We considered RD as a reference area since it is one of the largest PAs remaining in the Atlantic Forest of southeastern Brazil, with a diverse mammal community, including jaguars, mountain lions, tapirs (*Tapirus terrestris*) and giant armadillos (*Priodontes maximus*) [45, 46]. Although jaguars, tapirs and giant armadillos are absent in the other PAs, mountain lions can be detected in SB, SS, FMA and FM (Paschoal et al., in prep.).

### Sampling design

We used a standardized camera trap protocol to detect ocelots in the six reserves. Cameras were set to operate for 24 hours with an interval of five minutes between photos. Reserves were sampled for 80 consecutive days in each season (dry: April-September; wet: October-March).

In each study area, we selected 20 random sampling points (camera locations) from satellite images using ArcGIS 9.2 [49]. We distributed camera locations to ensure that at least one trapping station was located in a circular area equivalent to the smallest known home range of ocelots (76 ha; [50]). Any two adjacent trapping stations were up to 1 km apart, thus maximizing the probability of recording every individual present in the area. In the field, camera locations were placed as close as possible to the predetermined coordinates, usually within 50 m or 100 m, but preferentially placed along game trails, human paths, or unpaved roads because ocelots use these as travel routes [37–39]. We recorded the actual camera location using a GPS unit.

We installed camera traps in pairs to obtain simultaneous recording of the right and left sides of ocelots, allowing for individual identification. Because we only had ten cameras, we randomly moved pairs of cameras among sampling locations. We left cameras in place for 20 consecutive days before moving them to another five random points in the reserve, until all 20 points were sampled (total of 80 days). When we moved cameras, we also changed film and batteries. The total sampling effort, considering the pair of cameras at each location as a single sampling unit, was 800 camera trap-days in each reserve (400 camera trap-days /season).

### Estimating abundance, density and detection probability

We individually identified ocelots by stripe patterns on flanks, which are unique among individuals. Sex was determined by observation of genitals and the presence or absence of testes were used to distinguish between males and females. From these observations, we developed encounter histories for the 80 days of sampling in each season in each reserve depending on whether each individual was detected (1) or not (0). We collapsed our 80 days into groups of ten days (i.e., each individual encounter history contained eight occasions) in order to increase detection probabilities and improve estimates, as suggested by previous studies with elusive carnivores [51, 52]. We included sex as an individual covariate and used the Huggins closed capture model [53, 54] in Program MARK [55] to estimate abundance.

We mapped the land cover types by interpreting and classifying Landsat 5 images of each sampled area, using the technique of supervised classification and a maximum similarity algorithm in program ERDAS Image 8.4 [56]. We calculated the minimum convex polygon (MCP) formed by the outer sampling points in each reserve, which covered on average 910.6 ha (range 433.8 to 1,334.5 ha; Table 1). We added an additional buffer of about 3 km based on the mean maximum distance movement (MMDM; [57]) by ocelots detected in all reserves (Table 1). Inside this area (MCP + MMDM buffer) we calculated the proportion of forest and road network coverage (composed mainly by unpaved roads) in each reserve. To check if the proportion of forest inside the MPC + MMDM buffer accurately represented the amount of forest available in the larger landscape around the sampled areas, we mapped the proportion of forest inside an area of 10,000 ha centered around the MPC centroid of each reserve. This fixed area was large enough to accommodate the MPC + MMDM buffer. After that we performed a Pearson Correlation test between the proportion of forest mapped inside the MPC + MMDM buffer and inside the 10,000 ha area and found that both were highly correlated (r = 0.99). From this, we assumed that the proportion of forest inside the MPC + MMDM buffer accurately represented the amount of forest in the surrounding landscape. We used these predictor variables (i.e., covariates) for the analyses.

**Table 1:** Area covered by camera traps (minimum convex polygon—MCP—area), buffer area and effective trapping areas (ETA) based on two distances (MMDM = 2,718.61 m and ½ MMDM = 1,359.31 m) derived from camera traps in six Atlantic Forest reserves in southeastern Brazil.

Reserve	MCP (ha)	Buffer Area (ha)		ETA (ha)			
		MMDM	½MMDM	Total Area (MMDM)	Total Area (½MMDM)	Forest Area (MMDM)	Forest Area (½MMDM)
Fazenda Macedônia Reserve	1,073.32	5,910.70	2,374.68	6,984.02	3,448.00	429.48	429.48
Feliciano Miguel Abdala Reserve	754.05	5,545.87	2,192.08	6,299.92	2,946.13	2,237.29	1,450.65
Mata do Sossego Reserve	433.83	4,785.97	1,812.05	5,219.80	2,245.88	2,461.71	1,454.59
Serra do Brigadeiro State Park	1,334.51	6,309.67	2,574.25	7,644.18	3,908.76	3,974.50	2,343.11
Sete Salões State Park	980.41	6,119.87	2,479.44	7,100.28	3,459.85	3,781.25	2,193.14
Rio Doce State Park	830.97	5,481.00	2,159.95	6,311.97	2,990.92	3,544.83	2,074.27

We also considered the size of each reserve for the analyses as well as the number of free-ranging domestic dogs photographed in each reserve (i.e., the number of individuals that could be uniquely identified). We identified dogs based on their specific phenotypic differences and pelage coloration [29]. Finally, we considered the presence of both top predators (jaguar and mountain lion), which were detected only in the largest reserve (RD). Before using these covariates in our analysis, we tested for correlation among them using a Pearson Correlation Matrix, which indicated that none of the variables were highly correlated (|r| ≤ 0.50 in all cases).

We used four variables (percent of forest area, reserve size, number of free-ranging domestic dogs, and presence of both top predators; Table 2) in a variance components analyses in Program MARK [55]. We used a variance components analyses to focus on explaining the biological process variance (*δ* ^*2*^), which should not be confused with the sampling variance of ocelot abundance estimates [58, 59]. We estimated the percent of ocelot abundance variation explained by each variable. However, models from this analysis could not be compared using a model selection approach (e.g., AIC) because abundance ($\hat{N}$) is not in the likelihood in Huggins models. Therefore, we ran a mean model (intercept only) to obtain an overall estimate of process variance for each season. We then constructed additional models including each of these four variables alone for each season. We interpreted the resulting difference between the overall process variance (intercept only) and the process variance of a particular variable model as the amount of process variance explained by the variable. We also calculated the proportion of the biological variation explained as the difference divided by the overall process variance for each variable in each season.

**Table 2:** List of covariates used to model the variation in detection probability of ocelots among reserves, specifically the percentage of land covered by road networks and Forest Area, percentage of cameras installed on unpaved roads, the number of dogs detected in the reserve, reserve size and the presence of both Top Predators. Forest Area, Number of Dogs, Reserve size and Presence of both Top Predators were also used to model the process variance in abundance estimates of ocelot populations in six Atlantic Forest reserves in southeastern Brazil.

Reserve	Road Network Coverage (%)	Cameras Installed on Unpaved Roads (%)	Forested Area (%)	Number of Free-Ranging Domestic Dogs	Reserve Size (ha)	Presence of both Top Predators
Fazenda Macedônia Reserve	2.64	55.00	6.15	18	560	No
Feliciano Miguel Abdala Reserve	1.27	59.09	35.5	47	958	No
Mata do Sossego Reserve	0.14	0.00	47.14	9	134	No
Serra do Brigadeiro State Park	0.62	0.00	51.98	6	14,985	No
Sete Salões State Park	0.00	3.85	53.21	16	12,520	No
Rio Doce State Park	0.65	35.00	56.12	0	35,970	Yes

We calculated ocelot density by dividing $\hat{N}$ by the effective trapping area (ETA) in each reserve (Table 1). However, the estimated abundance of ocelots ($\hat{N}$) in one small reserve (FMA) was not reliable because we only recorded a single ocelot in each season and detection probabilities were very low (see Results). When the detection probability for rare and elusive carnivores is low (≤ 0.10) and each individual in the population is detected less than 2.5 times, the Huggins model has difficulty estimating abundance accurately [60]. Therefore, we used the observed abundance of ocelot to estimate density in FMA. We considered four different levels of ETA to estimate ocelot density (Table 1): MMDM buffer + MCP; ½ MMDM + MCP, and actual forest area within each of these previous levels of ETA. We considered forest area in calculating ocelot density because ocelots are considered a forest dependent species [24,40, 61]. Although MMDM has been considered a more accurate approach than ½ MMDM for estimating the area effectively sampled by cameras [34,62, 63], we also used the latter for two reasons. First, to make comparisons with other studies. Second, given the size of our MCPs, we judge the ½ MMDM may portray more faithfully the area of influence around the camera traps [62]. In one small reserve (MS), for example, the MMDM was almost ten times larger than the area sampled by cameras (MPC; Table 1) and, therefore, the MMDM may underestimate the ocelot density for this reserve. We calculate the polygons, buffers, and ETA using ArcGIS 9.2 [49].

Additionally, we modelled detection (*p*) and recapture (*c*) probabilities to estimate abundance ($\hat{N}$) for each season in each reserve. We considered detection structures with the effects of behavior (trap shy), sex (male vs female), season (dry vs wet), presence of both top predators (reserve with both predators -largest reserve; RD- vs other reserves; Table 2), landscape features (percent of forest area, percent of road network coverage and reserve size), PAs (or reserves), number of free-ranging domestic dogs and percent of cameras installed on unpaved roads (Table 2).

### Model selection and assumptions

We considered detection probabilities structures with all possible additive combinations of reserve (or covariates associated with each reserve), trap effect, season, and sex. We used Akaike's Information Criterion adjusted for small sample size (AICc), the relative AICc difference among models (ΔAICc), and associated model weights (AICc weights) to assess strength of candidate models [64]. This strategy resulted in a balanced model set and allowed us to calculate the cumulative AICc weights for each predictor variable [65]. Because of model selection uncertainty, we calculated model-averaged estimates of detection probability and abundance [64].

We examined violations of assumptions for closed population capture-recapture models [66]. We used the median $\hat{c}$ goodness-of-fit approach in Program MARK [67], which indicates no overdispersion (or independence among the sampled ocelots) when the $\hat{c}$ value is close to “1”. Our models assume that the population is closed geographically–no movement on or off the study area–and demographically–no births or deaths [66]. We tested for closure using the POPAN model in Program MARK, which allowed us to analyze the survival (*phi*) or egress (1 –*phi*) and ingress rates (*pent*) among capture occasions [68]. Using ΔAICc we compared models in which *phi* and *pent* parameters were fixed as “1” and “0” respectively (i.e., no egress or ingress) to models that allowed egress and ingress to vary to assess whether closure was achieved.

## Results

We did not detect overdispersion ($\hat{c}$ = 1.06 with 95% CI = 0.90–1.23) and our closure test revealed no violation (ΔAICc of the model without closure = 3.00).

The largest State Park (RD) and one small private reserve (FM) had the highest abundance and density estimates of ocelots (Table 3). Another small private reserve (FMA) had the lowest abundance and density estimates of ocelots among all reserves (Table 3) and one medium-sized reserve (SS) had the lowest abundance and density estimates of ocelots among the State Parks; no ocelots were detected there during the wet season (Table 3). When we look at the confidence intervals, however, we noticed that abundances and densities were similar among all areas, except for RD (Table 3).

**Table 3:** Abundance and density estimates for ocelots derived from camera-trap studies conducted in six Atlantic forest reserves, southeastern Brazil.

**Reserve**	Season	Abundance (±95% CI)	Density (ocelots/km^2^± 95% CI)			
			MMDM	½ MMDM	Forest MMDM	½ Forest MMDM
Fazenda Macedônia Reserve	Dry	5.04 (4.65–5.42)	0.07 (0.07–0.08)	0.15 (0.14–0.16)	1.17 (1.08–1.26)	1.17 (1.08–1.26)
	Wet	4.04 (3.62–4.46)	0.06 (0.05–0.06)	0.12 (0.11–0.13)	0.94 (0.84–1.04)	0.94 (0.84–1.04)
Feliciano Miguel Abdala Reserve	Dry	1	0.02	0.03	0.05	0.07
	Wet	1	0.02	0.03	0.05	0.07
Mata do Sossego Reserve	Dry	3.20 (2.18–4.22)	0.06 (0.04–0.08)	0.14 (0.10–0.19)	0.13 (0.09–0.17)	0.22 (0.15–0.29)
	Wet	1.07 (0.48–1.67)	0.02 (0.01–0.03)	0.05 (0.02–0.07)	0.04 (0.02–0.07)	0.07 (0.03–0.12)
Serra do Brigadeiro State Park	Dry	3.49 (1.79–5.19)	0.05 (0.02–0.07)	0.09 (0.05–0.13)	0.09 (0.05–0.13)	0.15 (0.08–0.22)
	Wet	4.70 (2.59–6.82)	0.06 (0.03–0.09)	0.12 (0.07–0.17)	0.12 (0.07–0.17)	0.20 (0.11–0.29)
Sete Salões State Park	Dry	2.21 (1.16–3.26)	0.03 (0.02–0.05)	0.06 (0.03–0.09)	0.06 (0.03–0.09)	0.10 (0.05–0.15)
	Wet	0	0	0	0	0
Rio Doce State Park	Dry	8.39 (5.28–11.51)	0.13 (0.08–0.18)	0.28 (0.18–0.39)	0.24 (0.15–0.33)	0.41 (0.26–0.56)
	Wet	8.51(5.26–11.76)	0.14 (0.08–0.19)	0.29 (0.18–0.39)	0.24 (0.15–0.33)	0.41 (0.25–0.57)

Reserve size, presence of both top predators and number of free-ranging domestic dogs all contributed to explaining variance of ocelot abundance (Table 4); ocelot abundance responded positively to reserve size and to presence of both top predators and negatively to abundance of free-ranging domestic dogs (Table 4). Further, the amount of variance explained by each of these variables varied seasonally (Table 4). The precision of these variance estimates were low (e.g., overlapping confidence intervals), suggesting that the differences in variance explained, both among variables and between seasons, should be considered with care.

**Table 4:** The percent of biological process variation in ocelot abundance explained by four reserve variables among six Atlantic Forest reserves in southeastern Brazil. Negative process variances were considered zero. See Methods for details.

Variables	Dry Season			Wet Season		
	δ^2^ Variance (±95% CI)	Beta Values (±95% CI)	% of Variation Explained	δ^2^ Variance (±95% CI)	Beta Values (±95% CI)	% of Variation Explained
Intercept only model	4.96 (1.62–32.87)	3.61 (1.75–5.47)	-	7.33 (2.03–68.25)	3.53 (1.04–6.01)	-
Reserve Size	3.05 (1.02–26.19)	0.1x10^-3^ (-0.3x10^-5^–0.3x10^-3^)	38.59	1.34 (0.39–19.46)	0.2x10^-3^ (0.8x10^-4^–0.3x10^-3^)	81.73
Presence of both Top Predators	2.11 (0.73–17.76)	4.81 (1.08–8.53)	57.47	3.19 (0.87–47.81)	5.34 (0.65–10.04)	56.50
Number of Domestic Dogs	3.33 (0.95–30.86)	-0.09 (-0.19–0.01)	32.90	5.74 (1.46–88.41)	-0.09 (-0.23–0.04)	21.63
Percent of Forest	5.57 (1.91–56.89)	-0.4x10^-2^ (-0.12–0.11)	0	8.38 (2.53–143.52)	0.03 (-0.11–0.18)	0

**Fig 2:**
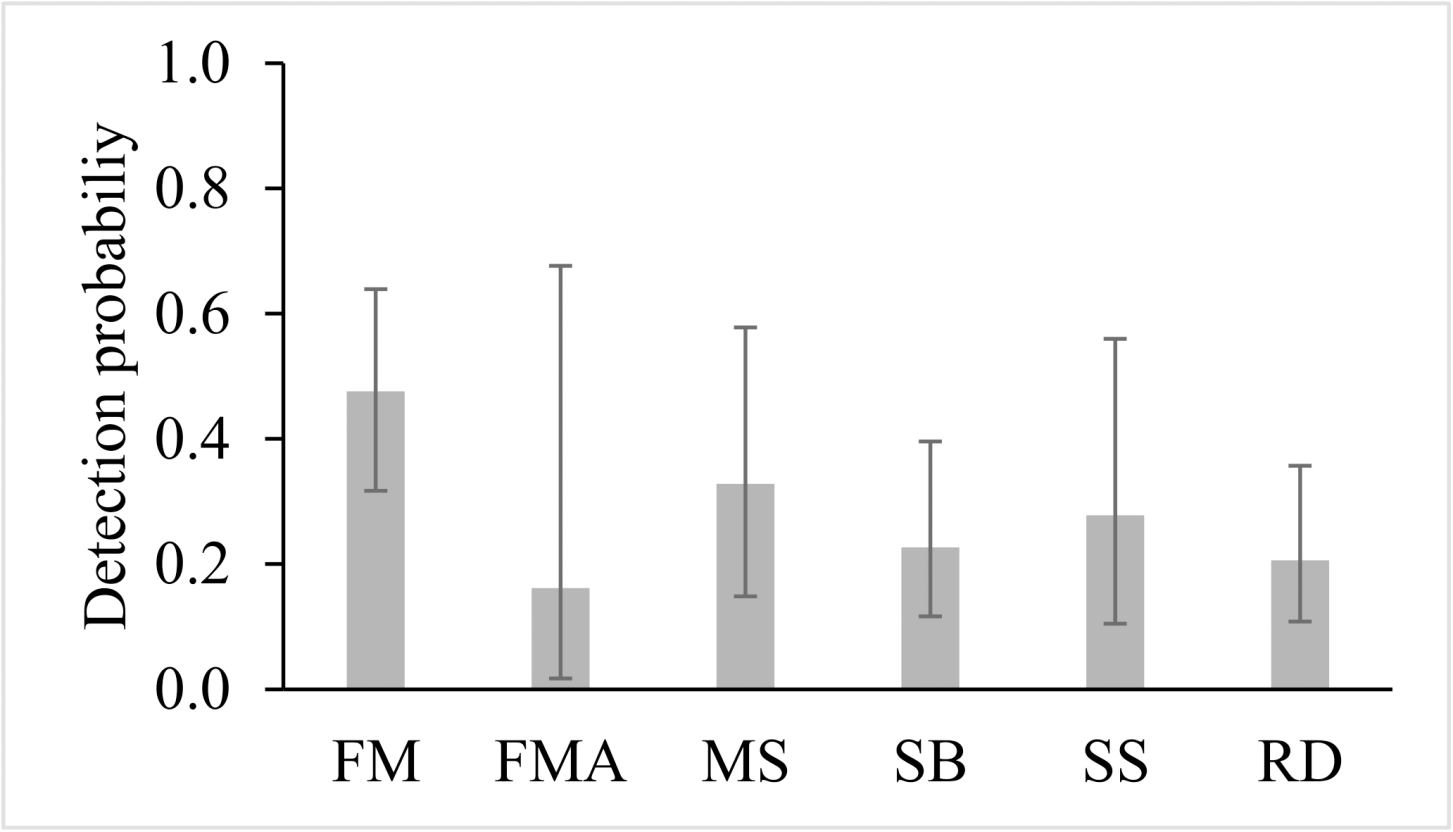
Model-averaged estimates of ocelot detection probabilities (*p*; ± 95% CI) in six Atlantic Forest reserves, southeastern Brazil. FM = Fazenda Macedônia Reserve; FMA = Feliciano Miguel Abdala Reserve; MS = Mata do Sossego Reserve; SB = Serra do Brigadeiro State Park; SS = Sete Salões State Park; RD = Rio Doce State Park.

Overall, the most parsimonious model in our candidate set indicated that the detection probability of ocelots varied among reserves (Table 5). Based on this model, detection probability of ocelots was higher in two small reserves (FM and MS), and lower in one small reserve (FMA) and in the largest reserve (RD; Fig 2). Of the reserve covariates used to model detection, the percent of forest was the only covariate that had more influence (cumulative AICc weights = 39.37%) on ocelot detection; the percent of forest had a negative relationship (β = -0.02 ± SE 0.01) with ocelot detection (Table 6). As expected, detection probability of ocelots was lower in more forested reserves, such as RD (Table 2; Fig 2), and higher in reserves with a lower proportion of forest cover, such as FM and MS (Table 2; Fig 2 **)**. The detection probability of ocelots in FM, for example, was more than two times higher than in RD (Fig 2), which has the highest forested area among all reserves (Table 2), but precision was low (large confidence intervals) due to small sample sizes (Fig 2). Although behavior, seasonality and sex had some influence on ocelot detection, they had low cumulative AICc weights (< 35%; Table 6). Road network coverage, reserve size, presence of both top predators, percent of cameras installed on unpaved roads and number of free-ranging domestic dogs had, respectively, the lowest cumulative AICc weights (< 6%) among the variables tested (Table 6).

**Table 5:** Model selection results for variables expected to influence ocelot detection probability in six Atlantic Forest reserves in southeastern Brazil. Only models with an AICc weights ≥ 0.01 are presented here.

Model^*^	AICc	ΔAICc	AICc Weights	Parameters	Deviance
p(Reserve) = c(Reserve)	353.86	0.00	0.17	6	341.58
p(Reserve) c(Reserve)	354.63	0.77	0.11	7	340.25
p(Forest) = c(Forest)	354.77	0.91	0.11	2	350.73
p(Forest+Sex) = c(Forest+Sex)	355.66	1.80	0.07	3	349.58
p(Forest+Season) = c(Forest+Season)	355.92	2.06	0.06	3	349.84
p(Reserve+Season) = c(Reserve+Season)	355.97	2.11	0.06	7	341.59
p(Forest) c(Forest)	356.16	2.30	0.05	3	350.08
p(Reserve+Sex) = c(Reserve+Sex)	356.25	2.39	0.05	7	341.87
p(Reserve+Season) c(Reserve+Season)	356.51	2.65	0.04	8	340.03
p(Forest+Sex) c(Forest+Sex)	356.78	2.92	0.04	4	348.64
p(Forest+Season+Sex) = c(Forest+Season+Sex)	356.78	2.92	0.04	4	348.65
p(Reserve+Sex) c(Reserve+Sex)	357.23	3.37	0.03	8	340.75
p(Forest+Season) c(Forest+Season)	357.33	3.47	0.03	4	349.20
p(Reserve+Season+Sex) = c(Reserve+Season+Sex)	357.55	3.69	0.03	8	341.06
p(Reserve size) = c(Reserve size)	358.72	4.86	0.01	2	354.68

^*^ The detection (*p*) and recapture (*c*) probability of ocelots modeled as function of: each reserve (Reserve); proportion of forest in each reserve (Forest); reserve size in ha (Reserve size); males and females (Sex) and; Season (Dry vs Wet). The equal signal (=) indicates that *p* and *c* have the same values for detection probability. The plus signal (+) means an additive effect between two or more tested variables.

**Table 6:** Cumulative AICc weights for variables used to model ocelot detection probabilities in six Atlantic Forest reserves in southeastern Brazil.

Variables	Cumulative AICc Weights (%)
Reserve	49.10
Forested Area (%)	39.37
Behavior Effect (trap shy)	34.62
Seasonality Effect (Dry vs Wet)	29.39
Sex Effect	29.17
Road Network Coverage (%)	5.20
Reserve Size (ha)	5.02
Presence of both Top Predators	1.11
% of Cameras Installed on Unpaved Roads	0.10
Number of Free-Ranging Domestic Dogs	0.08

## Discussion

Contrary to our expectations, we did not find higher abundance and density in fragments where the top predators were absent or rare. Rather, the presence of both top predators (jaguar and mountain lion) in the largest reserve (RD) correlated positively with an increased abundance of ocelots, especially during the dry season. Top predators may increase the area of forest by controlling the herbivory rates [69, 70], which might increase ocelot abundance because this species is dependent to canopy cover [24, 25]. In addition, high abundance and densities of territorial carnivores may positively correlate to prey density [71]. Jaguars, for example, were found only in RD and their presence may be related to a higher diversity of prey for this species, especially those of large body size, such as deer (*Mazama americana*) and collared peccary (*Pecari tajacu*) [72]. Our other study areas have less forest area and prey densities may not allow for ocelot, jaguar and mountain lion coexistence. In other words, the positive relationship between jaguars and ocelots might result from the fact that jaguar presence means better habitat for ocelots [28, 73] and for other carnivores. Jaguar abundance was positively related with mountain lion occupancy in the Cerrado of Central Brazil [74], and another study indicated that coexistence of both top predators are mediated mainly by food resources [75]. The presence of top predators, especially the jaguar in the Atlantic Forest, may be key in controlling the food chain and maintain prey availability in an ecosystem [9, 76].

Alternatively, jaguar occurrence may be positively correlated with ocelot abundance or density through the predation and/or harassment of potential ocelot competitors. We found a negative influence of dogs on ocelot abundance; the highest ocelot abundance was found in the largest reserve (RD) where we did not detect dogs. Therefore, the presence of jaguars may reduce the abundance of domestic dogs in a reserve via predation or interference competition [77]. Although domestic dogs did not exhibit a direct influence on the detection probability of ocelots, this exotic species may decrease prey availability [78] especially in small reserves, such as in FMA.

In a recent study, Paschoal et al. [29] found approximately 40 domestic dogs in FMA at a density about six times higher than that of ocelots, suggesting potential deleterious effects on ocelots. The current estimate of dog abundance in FMA seems to be almost two times higher (Paschoal et al., in prep.) than the abundances considered here (Table 2), which suggest that the influence of domestic dogs on the ocelot ecology could be stronger. For example, domestic dogs were also responsible for negatively affecting ocelot use (or distribution) in the same reserves of Atlantic Forest (Massara et al., in prep.) as well as the distribution of other felids in this biome, such as the margay (*Leopardus wiedii*) and the oncilla (*Leopardus tigrinus*) [79]. However, we do not know exactly the ecological mechanisms behind domestic dog occurrence that resulted in a decreasing on ocelot abundance in the studied reserves. These dogs are classified as rural free-ranging domestic dogs, which are owned or peripherally associated with human settlements but are not confined in a restrict area [80]. Although considered weak competitors, they may become important competitors and predators of wildlife because high densities of these dogs are subsidized by humans that live near natural habitats [78, 80]. Additionally, these dogs cause a variety of impacts apart from direct predation on wildlife, including the spread of disease [81]. At the same time, domestic dogs can exert more intrusive edge effects in more fragmented and smaller reserves, which are surrounded by a high density of human settlements and human-modified habitats, such as agricultural lands [80, 82]. In these reserves, these dogs can even form packs and explore natural areas, which make their impacts even higher upon medium- to large- sized mammals [29]. It may explain, for example, the high dog abundance and low ocelot abundance in smaller reserves, such as in FMA, which is dominated and surrounded by agriculture and human habitations. However, little is known about the variables that may indeed facilitate dog entrance in Brazilian natural areas or their direct effects on different species [29, 79, 83]. As domestic dogs are one of the most commonly recorded mammal species in the Atlantic Forest [29, 79, 84], managers of protected areas should start acting to mitigate or eliminate this hazard.

Reserve size also correlated positively with abundance of ocelots. Though it is difficult to compare densities among studies due to the lack of a standard sampling protocols and the inconsistency in quantifying the effective trapping area [62, 85], we found that larger areas usually have higher ocelot abundances and densities in the Atlantic Forest remnants (Table 7). Further, reserve size was negatively correlated (r = -0.92) with the edge ratio of each reserve, which suggests that our largest reserve (RD) may provide better quality of habitat for wildlife and suffer less edge effects, such as those exerted by the exotic species (e.g., domestic dogs).The proportion of forested area, however, did not positively correlate with ocelot abundance in the reserves. We suspect that it might be a reflection of one sampled reserve (i.e., Fazenda Macedônia; FM).

**Table 7:** Abundance and density estimates for ocelots derived from camera-trap studies conducted in Atlantic forest sites. Estimates are provided for two levels of buffers (MMDM, ½MMDM) according to their availability in each study. Ninety-five percent confidence intervals (95% CI) are presented, unless not included in a study.

Reserve	Country	Season	Sampling Effort (Trap—days)	Area (ha)	Abundance (± 95% CI)	Density (ocelots/km^2^)	
						MMDM	½MMDM
Yabotí Biosphere Reserve ^1^	Argentina	Wet	1,871	274,200	39 (35–54)	0.05	0.09
Iguazú National Park ^1^/ San Jorge Forest Reserve ^1^	Argentina/ Brazil	Wet	2,059	259,400	86 (75–111)	0.10	0.17
Iguazú National Park ^2^	Argentina	Both	1,631	170,000	55 (42–87)	0.13	0.20
Uruguaí Private Reserve ^2^	Argentina	Both	1,409	113,243	20 (18–35)	0.08	0.13
Ilha do Cardoso State Park ^3^	Brazil	Dry	585	15,100	6	0.21	-
Caraguatá Ecological Reserve ^4^	Brazil	Both	4,250	4,300	3.07	-	0.04
Feliciano Miguel Abdala Reserve ^5^	Brazil	Dry	450	957	2	0.16	0.35

^1^ [30]

^2^ [31]

^3^ [32]

^4^ [33]

^5^ [29]

Fazenda Macedônia had a relatively small size (560 ha), a high abundance and density of ocelots, and no jaguars (Tables 2 and 3). We believed that due to the proximity (15 km) of this reserve to the largest reserve (RD) and the existence of several smaller fragments connecting these two areas, the flow of ocelots among these fragments may be facilitated, making RD act as possible source of ocelots to FM. Young male ocelots (two or three years old), can disperse more than 10 km [13]. Further, FM has had potential prey species reintroduced, especially Galliformes and Tinamiformes birds [86], which may also attract predators, such as ocelots, to the area. However, longer-term studies and radio-tracking approaches are needed to test this hypothesis. At the same time, the high estimates of ocelot density in FM obtained using some buffers (i.e., Forest MMDM and ½ Forest MMDM; Table 3) relies on the fact that this area has the smallest proportion of forest among all reserves (Table 2), which may inflate the ocelot density through a mathematical artifact.

Although we did not detect closure violations, detecting such violations is difficult with small data sets. If the ocelot population is open then we are technically estimating a super-population (i.e., all individuals that use the sampled area during sampling; [68]). A super-population definition also aligns with potentially high turnover of ocelots among occasions and seasons, especially inside small or medium-sized fragments. In one small reserve (FMA) for example, we detect just one different individual in each season and no ocelots were recorded in one medium-sized reserve (SS) during the wet season. Further, in FMA the ocelots were only detected in a single occasion. The super-population concept may imply the existence of a metapopulation dynamic among fragments [87], reinforcing our suggestion of a flow of ocelot individuals between the largest reserve (RD) and one small reserve (FM).

Ocelots of different sex may have different home ranges [22,31, 34], and ranges may vary by season [34, 88]. Ocelots may use large trails or unpaved roads to move around the landscape [37–39]. However, we did not find strong support for these variables affecting detection probability of ocelots. Although the proportion of forest had just some influence (AICc weights = 39.37%) on ocelot detection, it was the reserve variable that best explained the variation in ocelot detection. Low detectability in more forested areas may relate to large ocelot home ranges in these areas, where individuals have a larger amount of forested area to use. Conversely, in areas poorly covered by forests, ocelots may have smaller home ranges (i.e. Bolivia; [62]) and concentrate travel (about 3 to 7 km per night) in a smaller area to attain their daily energy requirements [22, 24, 89], which can increase their detection probabilities. This reasoning however does not explain our results in one small reserve (FMA), which has the second lowest proportion of forest among all sampled areas (Table 2) but the lowest detection probability (Fig 2). We believe that some other variables that we did not measure in this present study may better explain the variation in ocelot detection probability among reserves and should be investigated in future studies. Some obvious possibilities that are known to affect mammal populations includes degree of surveillance or poaching pressure [3, 90]. We refrain to speculate about these, given that an accurate assessment of such effects are lacking for our six reserves.

We do have data on the immediate surrounding landscapes of our reserves. One of our small and least forested reserve (i.e., FM) for example, is surrounded by eucalyptus, which may be used constantly by ocelots as travel routes to move between native habitats within or outside the reserve [91]. Because ocelot is a forest dependent species [25,61, 92], it may uses eucalyptus more often than open habitats (e.g., pasture or croplands) to find native habitats (e.g., native forest). Therefore, reserves surrounded by more permeable matrices may have higher ocelot detection than areas surrounded by more inhospitable habitats (e.g., pasture around FMA).

Overall, our findings suggest that top predators, especially the jaguar, seem to act as an umbrella species for ocelots and other sympatric mesocarnivores [73] and that ecological processes that are detrimental to top predators may also be detrimental to ocelots. By protecting top predators we may also protect other species, such as ocelots. Indeed, top predators have been target by conservation initiatives to protect entire communities in different ecosystems [76]. Although our data show that the ocelot is able to inhabit smaller reserves, the lower densities (except for FM) indicate that these reserves might represent poor habitats. These results corroborates other authors working on the effects of forest fragmentation in the Atlantic forest, which show that only large fragments in the range of 20,000 ha or more can sustain viable populations of medium to large sized mammal species [6, 7, 27].

Low densities in small fragments translates to small populations with low viability. In the USA, for example, only two known isolated ocelot populations occur in southern Texas. For these isolated populations, conservation concerns include loss of dense forest habitat, mortality from vehicle-collisions, and genetic drift [93]. A habitat-based population strategy was adopted for the recovery efforts of these populations [92, 93]. The long-term recovery strategy included the restoration of ocelot habitat and the establishment of a dispersal corridor between ocelot breeding populations [92]. Whether increased connectivity will be able to overcome genetic drift or the reduction in the genetic diversity is unknown [94–96]. Unfortunately, a similar situation may be occurring among the remnant ocelot populations in the Atlantic Forest. A recent study found the first report of a unilateral cryptorchidism (i.e., the absence of one testis from the scrotum) in an wild adult ocelot, an inherited condition linked to low genetic variability in inbred wild cats [97]. This finding is especially concerning because it comes from the largest of our study areas (RD, with 36,000 ha). Therefore, without increased connectivity, the outlook for ocelots in the Atlantic Forest may be pessimistic, a view also backed by others [30, 31].

## Conclusion and Recommendations

Our findings do not support the hypothesis of mesopredator release. Rather, our analyses indicate that presence of top predators and reserve size correlated positively with an increased abundance of ocelots in the Atlantic Forest reserves. The implementation of biodiversity corridors could protect and increase the current ocelot population in small Atlantic Forest fragments, reducing the isolation of small populations and augmenting structural and functional connectivity among forest patches. However, a better alternative might be based on improving connections via native vegetation and protection through the Brazilian Forest code (Federal Law number 12,651 from May 25, 2,012). Preliminary data of an ongoing project carried out in São Paulo state show, for example, that ocelots do inhabit areas of permanent protection (Áreas de Proteção Permanente—APPs), even when these are immersed in sugar cane or eucalyptus matrices [98]. According to the Brazilian forest code, these APPs protect mainly watercourses. Therefore, the possibility that these areas act like true corridors might indeed be real. We note that one small reserve (FM) and the largest reserve (RD) are linked by the Rio Doce River. Implementing the Forest Code law would therefore translate to increasing structural connectivity between these two protected areas via restoration of riparian forests along the Rio Doce River. Future studies should, investigate more closely these areas and their surrounding matrices in order to assess their use by ocelots.
